# Use of bioinformatic database analysis and specimen verification to identify novel biomarkers predicting gastric cancer metastasis

**DOI:** 10.7150/jca.58768

**Published:** 2021-08-13

**Authors:** Weimin Wang, Ke Min, Gaoyang Chen, Hui Zhang, Jianliang Deng, Mengying Lv, Zhihong Cao, Yan Zhou

**Affiliations:** 1 Department of Oncology, Yixing Hospital Affiliated to Medical College of Yangzhou University, Yangzhou University, Jiangsu, China.; 2 Institute of Combining Chinese Traditional and Western Medicine, Medical College, Yangzhou University, Jiangsu, China.; 3 Department of Oncology, The second People's Hospital of Taizhou City, Jiangsu, China.; 4 Department of Nursing, SuZhou Vocational Health College, Jiangsu, China.

**Keywords:** Gastric cancer, Metastasis, Prognosis, Biomarker, WGCNA

## Abstract

**Background:** Gastric cancer (GC) is a common gastrointestinal tumor, and its metastasis has led to a significant increase in the death rate. The mechanisms of GC metastasis remain unclear.

**Methods:** The differentially expressed genes (DmRs) and lncRNAs (DlncRs) of GC were selected from The Cancer Genome Atlas (TCGA) database. We applied the weighted gene co-expression network analysis (WGCNA) to construct co-expression modules related with GC metastasis. Gene ontology (GO) and Kyoto Encyclopedia of Genes and Genomes (KEGG) method analyzed the functional regions and signal pathways of genes in vital modules. DmRs-DlncRs co-expression network were drawn for finding out hub nodes. Survival analyses of significant biomarkers were analyzed by Kaplan-Meier (KM) method. Finally, the expressions of selected biomarkers were validated in cell lines and caner tissues by quantitative real-time PCR (qRT-PCR), in GC tissue microarray by Fluorescence *in situ* hybridization (FISH).

**Results:** 4776 DmRs and 213 DlncRs were involved the construction of WGCNA network, and MEyellow module was identified to have more significant correlation with GC metastasis. DmRs and DlncRs of MEyellow module were proved to be involved in the processes of cancer pathogenesis by GO and KEGG pathway analysis. Through the DmRs-DlncRs co-expression network, 7 DmRs and 1 DlncRs were considered as hub nodes. Besides, the high expression of TIMD4, CETP, KRT27, PTGDS, FAM30A was worse than low expression in GC patients survival, respectively; However, LRRC26 was opposite trend. FAM30A and TIMD4 were all significant biomarkers of GC survival and hub genes. Simultaneously, TIMD4, CETP, KRT27, PTGDS, FAM30A were increased in GC cell lines and tissues compared with GES-1 and normal tissues, respectively; the expression of LRRC26 was reduced in GC cell lines and tissues.

**Conclusion:** This study identified 6 genes as new biomarkers affecting the metastasis of GC. Especially, FAM30A and TIMD4 might be an effective marker for predicting the prognosis and a potential-therapeutic target in GC.

## Introduction

Gastric cancer (GC) is a common malignant tumor of the digestive system. The mortality rate of GC ranks second among all malignant tumors worldwide 1. With the diversification of treatment modes, the treatment methods for GC primarily include surgery, chemotherapy, radiotherapy, targeted therapy, and immunotherapy 2. Although these treatment methods could reduce the recurrence rate of GC, the 5-year survival rate of this cancer is still low 3. The occurrence and development of GC is a continuous, multistage, multifactor process. The pathogenesis of GC is complicated and involves genetic and epigenetic changes, such as protein-encoding genes, lncRNAs, and miRNAs 4. Therefore, it is particularly important to uncover effective biomarkers associated with GC metastasis to improve the overall survival (OS) of GC in this process.

In recent years, an increasing number of studies have focused on RNA sequencing (RNA-Seq), which is a rapidly maturing second-generation sequencing technology. The Cancer Genome Atlas (TCGA), as the world's largest public tumor database, provides an RNA-Seq platform that contains mRNA, lncRNA, and miRNA data for various cancers. With these sequencing results, we identified new biomarkers to predict tumor metastasis and improve OS through bioinformatic analysis. Weighted gene coexpression network analysis (WGCNA) is an important bioinformatic analysis method that can design clusters or modules of highly similar biomolecules and identify internal modular “hubs”, including mRNA, lncRNA, and miRNA 5-7. Furthermore, these modules and sample features were analyzed by WGCNA, which was able to investigate the mechanism underlying certain features 8. WGCNA was employed to construct coexpression modules to identify essential genes in human osteosarcoma 9. The WGCNA method was utilized to determine that SERP2, EFEMP2, FBN1, SPARC, and LINC0219 were recurrence‑related molecules and prognostic markers in colon cancer 10. However, studies employing WGCNA to investigate GC metastasis have not been reported.

In our study, we comprehensively analyzed RNA-Seq data of GC patients in the TCGA database and successfully identified a group of differentially expressed mRNAs (DmRs) and lncRNAs (DlncRs). After merging the DmRs and DlncRs of GC, we conducted WGCNA and module-trait relationship analyses to illustrate significant modules related to GC metastasis. Immediately, cell functional areas and signaling pathways of important modules were excavated by Gene Ontology (GO) and Kyoto Encyclopedia of Genes and Genomes (KEGG) analyses. A DmR-DlncR coexpression network analysis and a survival analysis of biomarkers in significant modules were performed. We chose 6 candidate biomarkers to be validated in GC cell lines and GES-1 cells, fresh GC tissues and normal tissues by quantitative real-time PCR (qRT-PCR). Finally, after combining the results of DmR-DlncR coexpression and survival curves, we used the GC population sample database to verify 2 candidate biomarkers by fluorescence *in situ* hybridization (FISH). This comprehensive analysis might provide potential biomarkers or therapeutic targets for future research investigating GC metastasis at the transcriptomic level.

## Materials and methods

### Study design and collection of datasets

**Figure 1:**
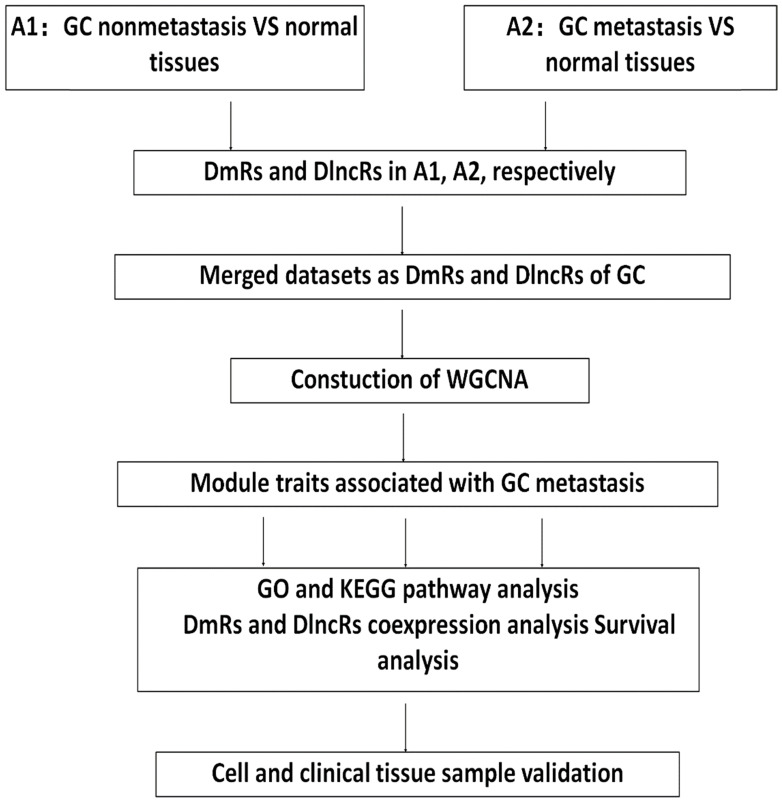
Flow chart of data processing, analysis and verification.

The design of this article is outlined in Fig. 1. The datasets on the expression of mRNAs and lncRNAs from GC patients were obtained from the TCGA database through the Illumina-HiSeq RNA-Seq platform. Excluding patients with other tumors or patients without metastatic clinical information, the mRNA and lncRNA expression data included 367 GC nonmetastasis samples, 27 metastasis samples and 35 normal samples.

### Identification of DmRs and DlncRs

The limma package of R was used to analyze the DmRs and DlncRs between GC metastasis, nonmetastasis and normal samples according to a false discovery rate (FDR) < 0.05 and a fold change (FC) > 2. These samples were divided into two groups: A1 (GC nonmetastasis vs. normal tissues) and A2 (GC metastasis vs. normal tissues). Then, we merged the datasets of DmRs and DlncRs of GC for WGCNA analysis.

### WGCNA analysis

Using WGCNA (version 1.61), the merged DmRs and DlncRs of GC were applied to construct coexpression modules and perform network analysis 11. First, we selected the soft threshold for network construction, which made the adjacency matrix a continuous value between 0 and 1. According to this method, the coexpression network conformed to the power law distribution and was closer to the actual biological network state. Second, the blockwiseModules function was employed to construct a scale-free network. The gene coexpression modules were constructed by module allocation analysis. We used the dynamic tree cutting algorithm to cut the clustering tree into branches to define these modules and assigned them to different colors for visualization 12. The module eigengene (ME) was calculated to represent the expression level of each module. The correlation between ME and clinical traits was calculated. Finally, we identified genes with significant differences for further analysis.

### Recognition of significant modules associated with GC metastasis

These module eigengenes (MEs) were the primary components of each gene module constructed by the WGCNA algorithm. The potential relevance of gene modules and clinical traits could be evaluated. The significant coexpression module related to GC metastasis was identified according to the traits of clinical characteristics in the GC metastasis and nonmetastasis groups. Module-trait relationships were calculated by Pearson's correlation tests. P < 0.05 was defined as a significant correlation. DmRs and DlncRs in significant modules were subjected to further analysis.

### Cell function and pathway enrichment analysis of significant modules

GO and KEGG pathway analyses of DmRs and DlncRs were performed according to Annotation, Visualization and Integrated Discovery of the database in significant modules. P < 0.05 was selected as the cut-off criterion that could identify important GO biological functions and KEGG pathway analysis results.

### Construction of DmR and DlncR coexpression networks and investigation of hub genes

We constructed DmR and DlncR coexpression networks based on nodes of significant modules to illustrate the relationship between DmR and DlncR. The key nodes (hub genes) were determined by the high intramodule connectivity of genes, which could represent the strength of connections with other modular genes. The coexpression networks and hub genes in vital modules were visualized and analyzed by Cytoscape software (Version 3.5.1) 13.

### Survival analysis

To detect the prognostic value of DmRs and DlncRs in significant modules, survival analysis was performed by the Kaplan-Meier (KM) method in the Gene Expression Profiling Interactive Analysis (GEPIA) database of TCGA 14. A total of 408 GC samples were included, which were divided into high-expression and low-expression groups according to the median value. P < 0.05 was considered to be significant.

### Cell culture and fresh GC tissue acquisition

GC cell lines (AGS, HGC27 and MKN45) and normal gastric mucosa cells (GES-1) were purchased from the Cell Bank of the Chinese Academy of Sciences, Shanghai Institute of Cell Biology (Shanghai, China). Cells were inoculated into RPMI-1640 medium (HyClone, Thermo Fisher Scientific Biochemical Products Co., Ltd., China) supplemented with 10% fetal bovine serum (Thermo Scientific HyClone, Logan, UT, USA). Cells were maintained at 37 °C in a humidified atmosphere containing 5% CO_2_. The 6 paired fresh tissues obtained through surgical resection were frozen in liquid nitrogen immediately and sent to the laboratory to for RNA extraction and RT-PCR detection.

### RNA extraction and qRT‑PCR analysis

RNA was extracted from GC cell lines, GES-1 cells and fresh tissue using an RNeasy Mini Kit (Invitrogen, Carlsbad, USA) according to the manufacturer's instructions. The purified RNAs were reverse transcribed to cDNA using the PrimeScript RT reagent Kit (Takara Biotech, Dalian, China). SYBR Green Real-Time qPCR analysis was performed using an Applied Biosystems 7500 Real-Time PCR System (Roche Applied Science, Penzberg, Upper Bavaria, Germany). The gene-specific sequences are listed in Table 1.

### GC database specimens and construction of tissue microarray (TMA)

A total of 380 patients underwent GC surgery on tissue chips from January 2010 to December 2013 in Jiangsu Province Yixing People's Hospital. All patients were followed up for at least 3 years. Overall survival (OS) was calculated from the date of surgery to the date of death or to the last follow-up.

The cancer tissues and corresponding adjacent tissues were used for TMA construction. The GC TMA included 760 cores. The construction of TMAs was undertaken by Shanghai Chip Super Organizing Chip Co., Ltd.

### Fluorescence *in situ* hybridization (FISH)

FISH probes directly labeled with fluorochromes are commercially available in red. The protocol was performed according to the FISH kit instructions (Ribobio, Guangzhou, China). The average optical density of immunofluorescence at each point was scanned and calculated by Image-Pro Plus 6.0 software (Media Cybernetics, lnc, Rockville, MD, USA). The average optical (AO) = cumulative optical density value (IOD)/pixel area of tissue (AREA). A higher AO value indicates a higher positive expression level.

### Statistical analysis

Prism 6.0, SPSS 21.0 or R software 3.5.1 were used for all data analyses. Student's t test was used to analyze the differences between two groups. Using the Kaplan-Meier method to draw survival curves, the log-rank test was used to compare survival differences. P < 0.05 was considered to be significant (*p < 0.05; **p < 0.001).

## Results

### Identification of DmRs and DlncRs

**Figure 2:**
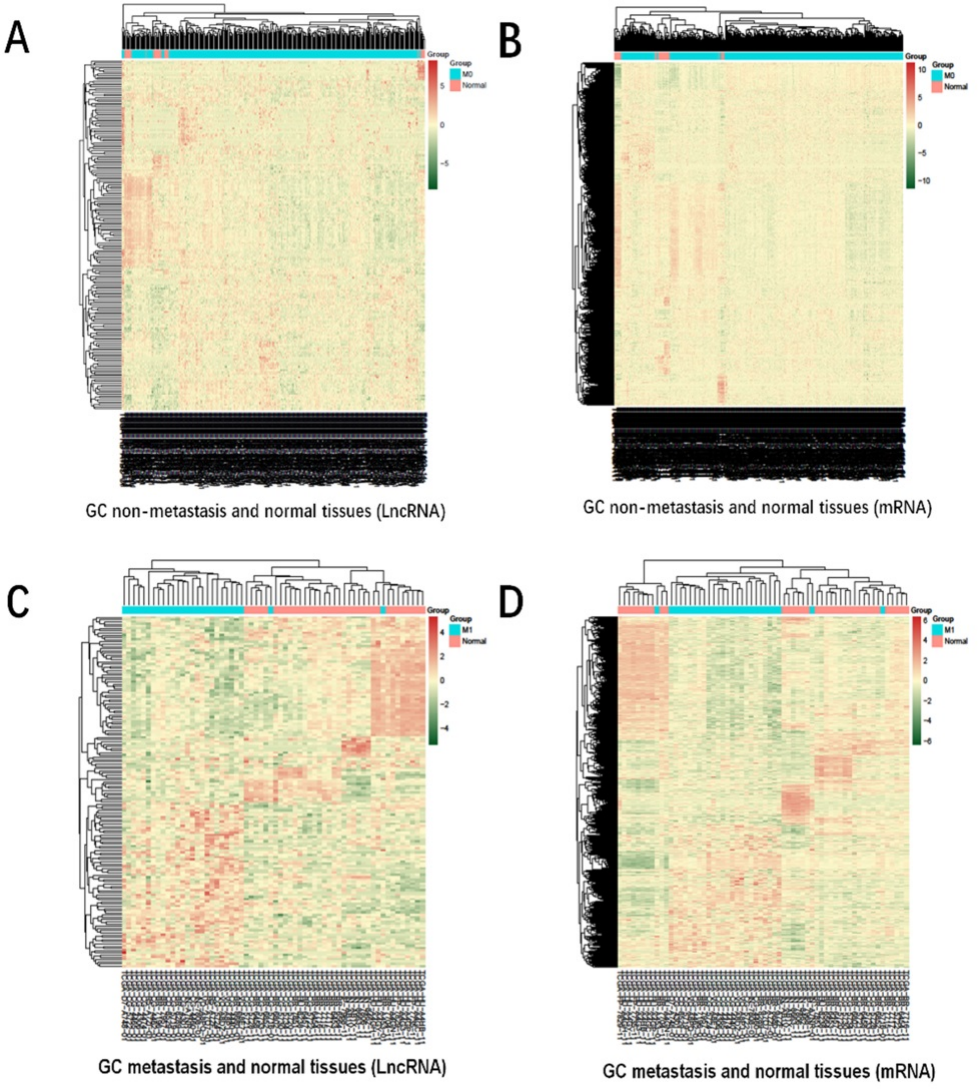
Heatmap for hierarchical cluster analysis of DmRs and DlncRs expression levels. **A, B:** GC nonmetastasis and normal tissues (A1); **C, D:** GC metastasis and normal tissues (A2). The red and green colors represent higher expression levels and lower expression levels of DmRs and DlncRs, respectively.

The heatmap of DmRs and DlncRs in GC metastasis and nonmetastasis groups is presented in Fig. 2. There were 4228 DmRs and 192 DlncRs in the GC metastasis vs normal group, while there were 3729 DmRs and 158 DlncRs in the nonmetastasis vs normal groups. Subsequently, we conducted the following analysis based on this part of the data.

### Coexpression network construction and key module identification

**Figure 3:**
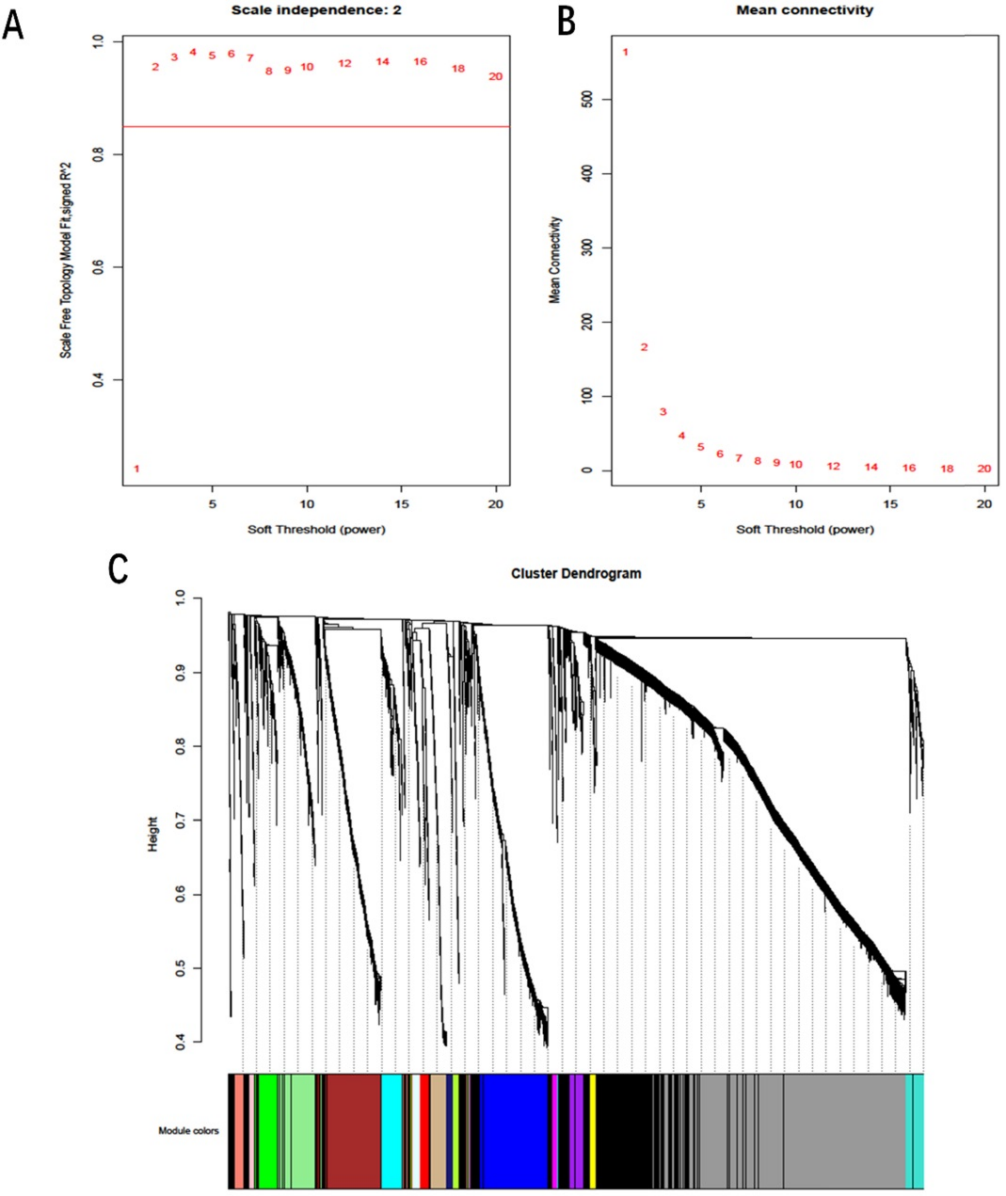
** A:** Scale independence. **B:** Mean connectivity analysis for various soft-thresholding powers. **C:** Clustering dendrogram of DmRs and DlncRs based on a dissimilarity measure (1-TOM), with dissimilarity based on topological overlap. Each color represents one coexpression module (gray represents unassigned DmRs and DlncRs).

A total of 4776 DmRs and 213 DlncRs were involved in the construction of the coexpression network by the WGCNA method. In this study, we utilized scale independence and mean connectivity analysis of modules (values from 1 to 20) to determine the soft threshold. The power of β = 2 was selected as the soft-thresholding parameter to construct a scale-free network (Fig. 3A, B). Placing the average linkage clustering of all DmRs and DlncRs with similar expression patterns into modules (Fig. 3C) could generate 18 modules with different colors. The distribution of DmRs and DlncRs among these modules is summarized in Table 2.

**Figure 4:**
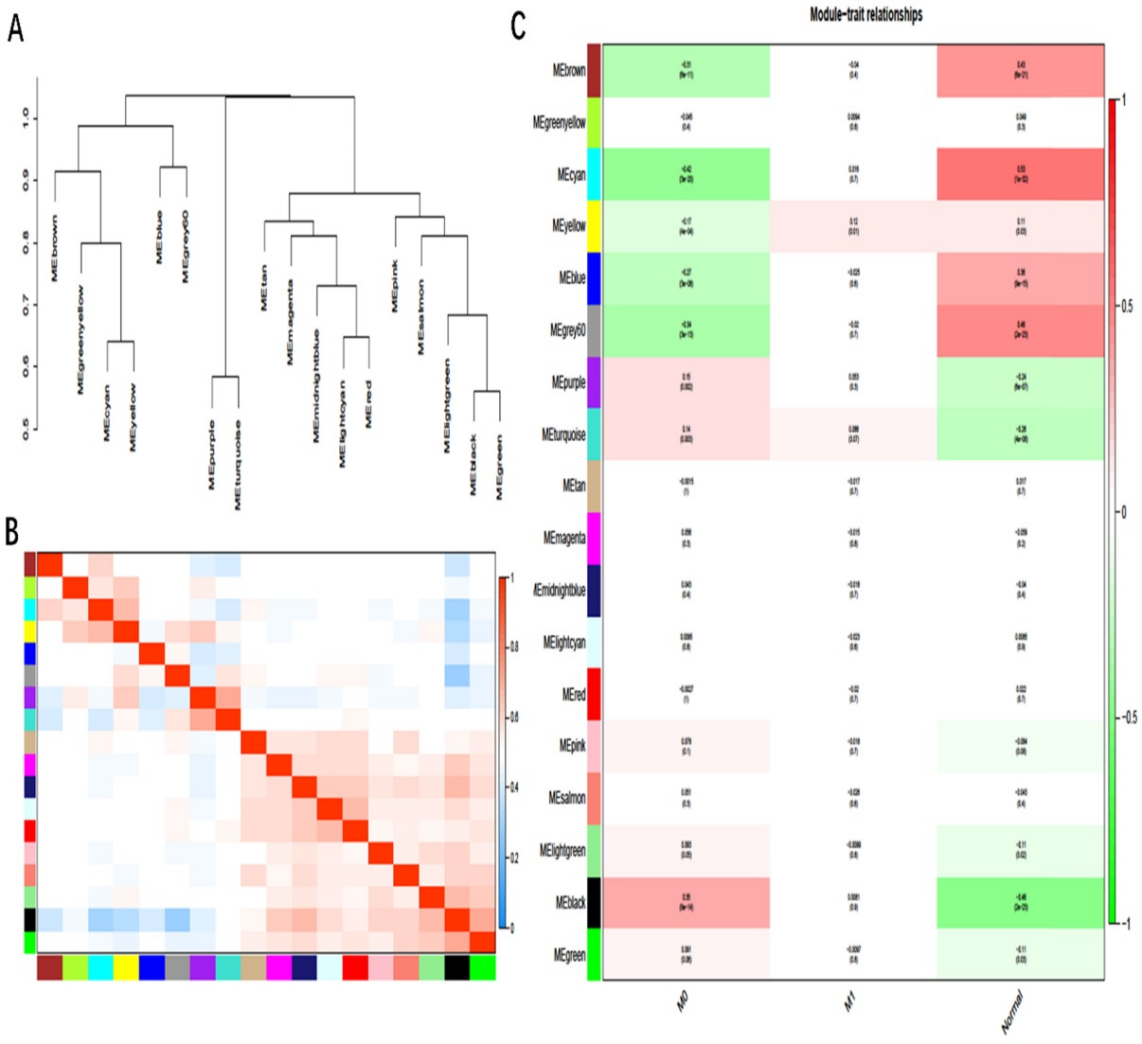
** A, B:** Hierarchical clustering of heat maps and adjacent modules of feature genes. **C:** Module-trait relationships: Each row corresponds to a module eigengene, column to a trait; the correlation coefficient and corresponding P-value was shown in the correlation between the gene module and the clinical trait. M0: cancer, nonmetastasis; M1: cancer, metastasis.

As in Fig. 4A and 4B, there were eigengene adjacency networks and hierarchical clustering dendrograms of the eigengenes. The MEyellow module associated with GC metastasis was identified by WGCNA analysis with P < 0.05 (Fig. 4C). There were 42 DmRs and 2 DlncRs in the MEyellow module, which was selected as the clinically significant module for further analysis.

### Functional area and signal pathway analysis of the MEyellow module

**Figure 5:**
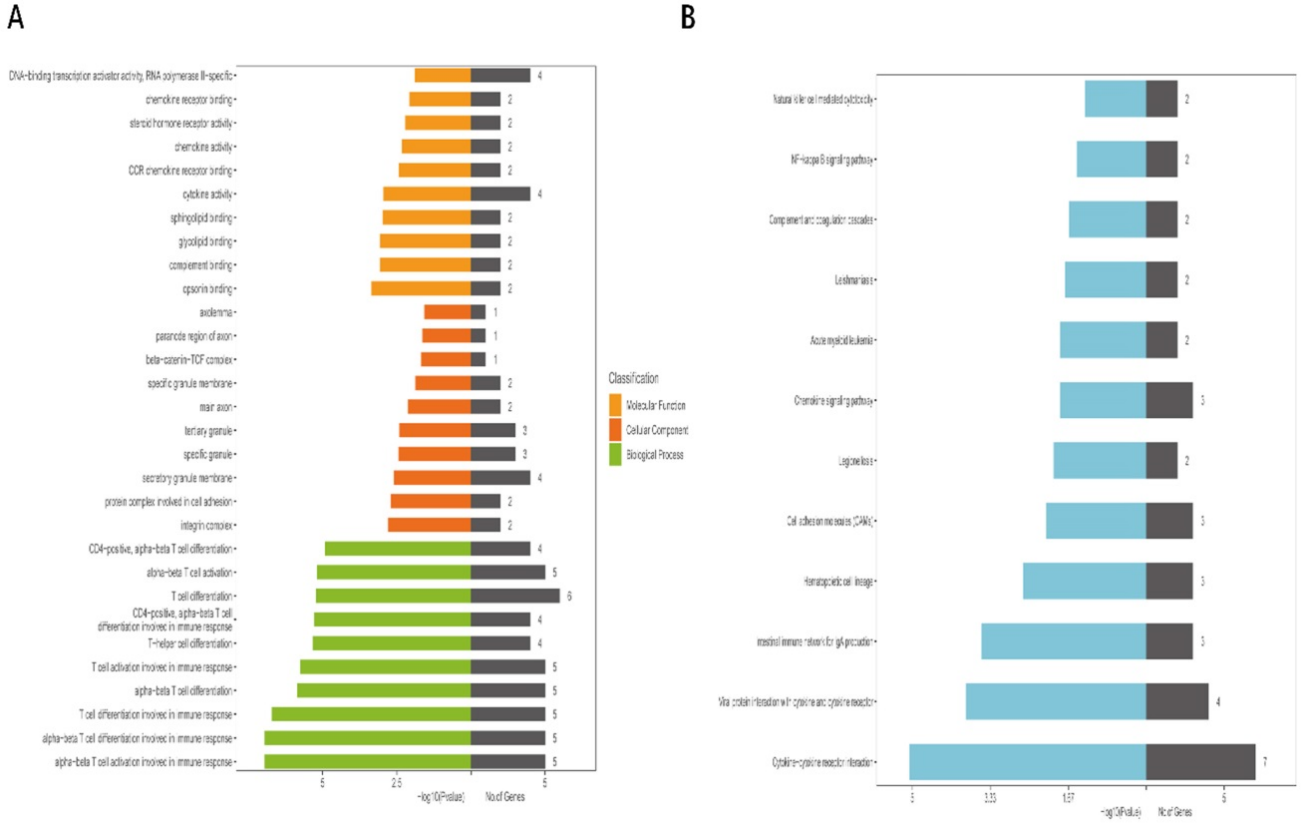
** A, B:** GO and KEGG pathway analysis of DmRs and DlncRs in MEyellow module.

We used GO analysis and KEGG pathway enrichment analysis to detect the underlying biological processes of DmRs and DlncRs. According to GO analysis, our results demonstrated that DmRs and DlncRs were primarily involved in opsonin binding, complement binding, glycolipid binding, sphingolipid binding in molecular function; the integrin complex, the protein complex involved in cell adhesion, the secretory granule membrane in cellular components, and alpha-beta T cell activation and differentiation in biological processes (Fig. 5A). KEGG pathway analysis indicated that these genes were primarily related to cytokine-cytokine receptor interactions, viral protein interactions with cytokines and cytokine receptors and intestinal immune networks for IgA production (Fig. 5B).

### Construction of the DmR and DlncR coexpression network and identification of hub genes

**Figure 6:**
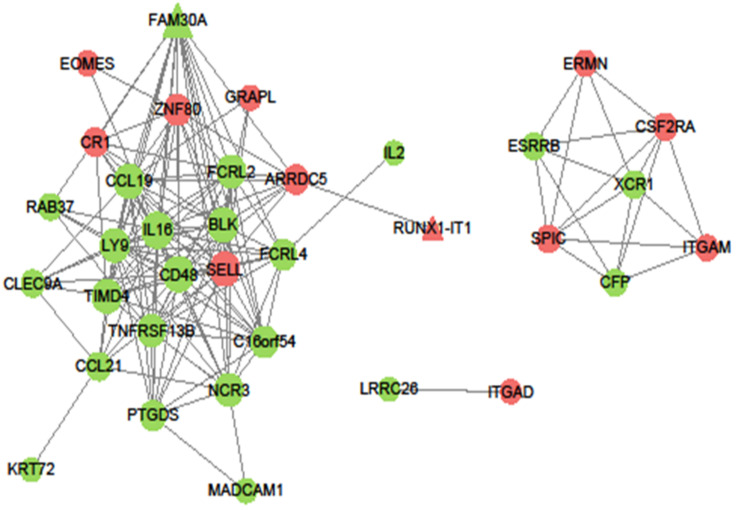
Coexpression pattern of DmR-DlncR in MEyellow module. The circular nodes indicate the DmRs, triangle nodes indicate DlncRs. Red represents upregulation, while green represents upregulation.

The DmRs-DlncRs coexpression network was constructed based on the correlation analysis in the MEyellow module (Fig. 6). The node had a higher degree, which played a more important role in this network among these networks. We determined that nodes with degrees greater than 15 were considered hub nodes. In this study, our results found that there were 8 hub nodes, including 7 DmRs (IL16, CD48, CCL19, SELL, BLK, LY9, and TIMD4) and 1 DlncR (FAM30A) (Table 3).

### Survival analysis of vital biomarkers and validation by qRT-PCR

**Figure 7:**
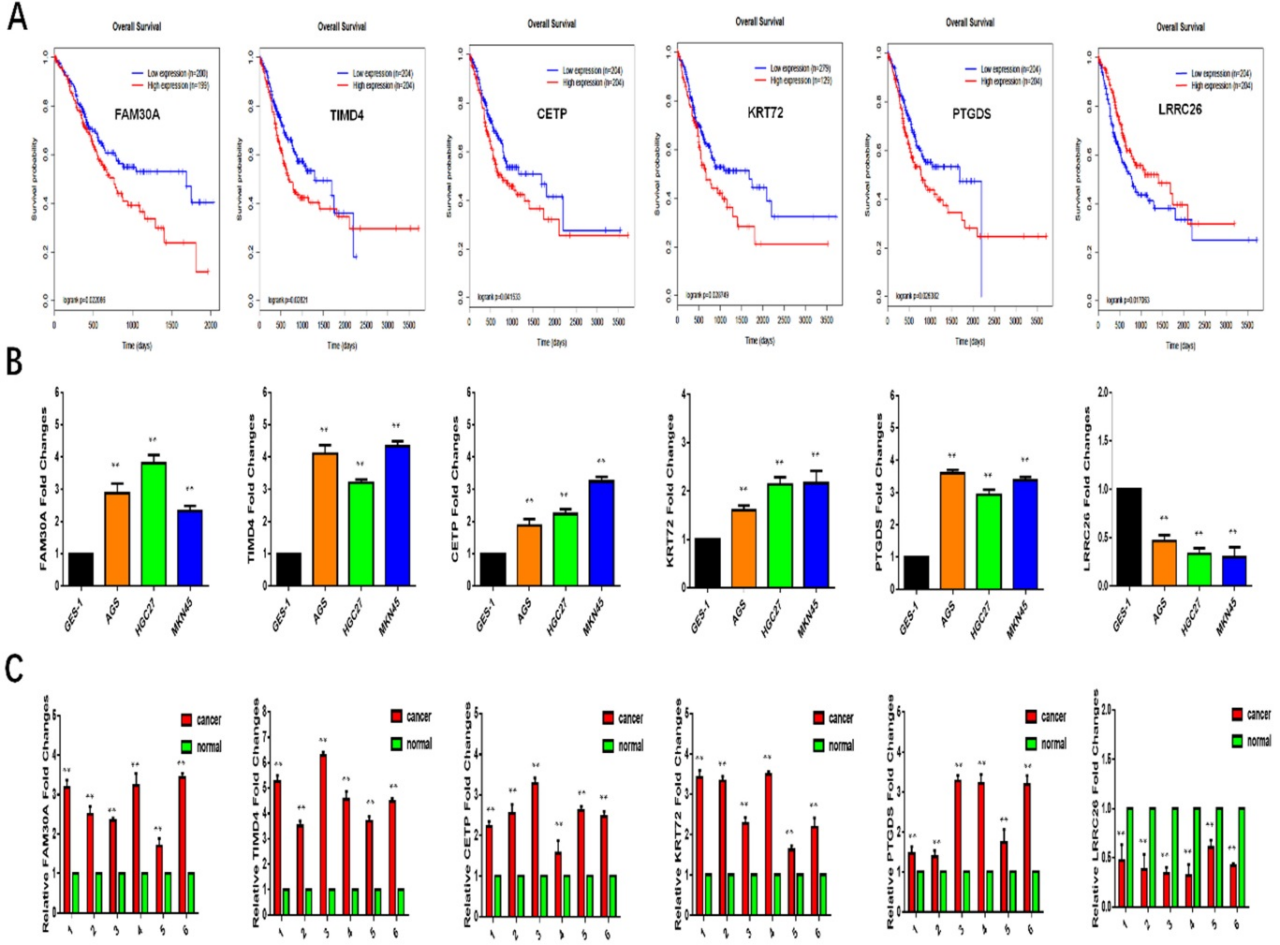
** A:** Survival analysis of FAM30A, TIMD4, CETP, KRT27, PTGDS, LRRC26. **B, C:** The expression of FAM30A, TIMD4, CETP, KRT27, PTGDS, LRRC26 in GC cell lines or tissues by qRT-PCR.

The DmRs and DlncRs in the MEyellow module associated with the survival of GC patients were analyzed by the Kaplan-Meier method. We found that 5 DmRs (TIMD4, CETP, KRT27, PTGDS, and LRRC26) and 1 DlncR (FAM30A) were associated with the survival prognoses of GC patients. High expression of TIMD4, CETP, KRT27, PTGDS, and FAM30A was worse than low expression regarding GC patient survival. However, LRRC26 exhibited the opposite trend (P < 0.05) (Fig. 7A).

To further study the role of these genes in GC metastasis, we applied qRT-PCR to verify their expression in cells and tissues. As shown in Fig. 7B and 7C, the expression levels of TIMD4, CETP, KRT27, PTGDS, and FAM30A were increased in GC cell lines and tissues compared with GES-1 and normal tissues (P < 0.05, respectively). Meanwhile, the expression of LRRC26 was reduced in GC cell lines and tissues. All these results indicated that the expression of these biomarkers was consistent with predicting the survival trend of GC prognosis.

### Validation of GC database specimens

Based on significant biomarkers of GC survival and hub genes, we found that FAM30A and TIMD4 could predict the prognosis of GC and were hub genes. Therefore, we selected these two molecules to be verified with GC database specimens.

**Figure 8:**
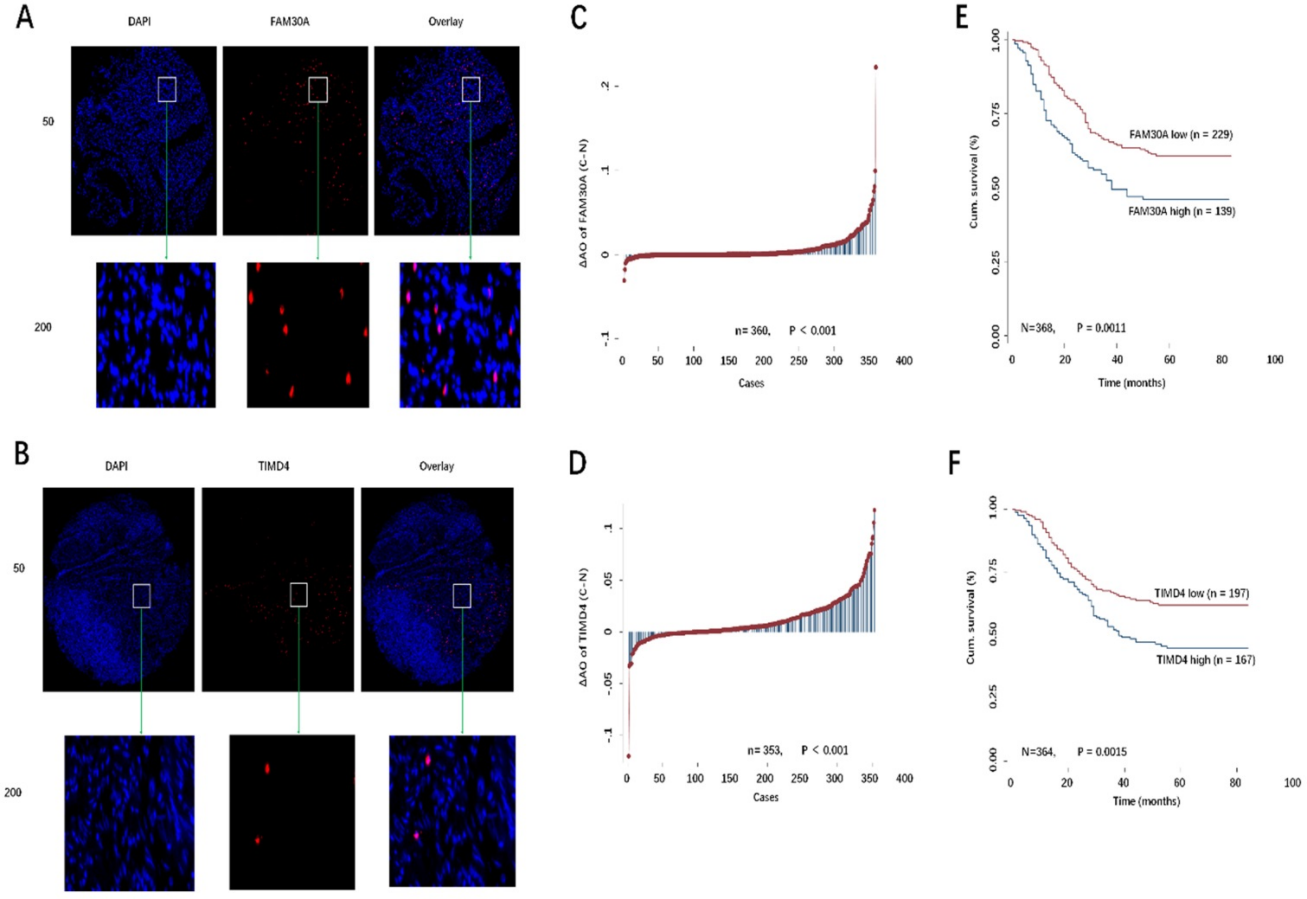
** A, B:** Representative images of FAM30A and TIMD4 FISH immunofluorescence staining in TMA were showed: top panel: original magnification, ×50; bottom panel: ×200. **C, D:** The different distribution of FAM30A and TIMD4 in GC compared with paired normal tissues in TMA. E, F: Kaplan-Meier curves of the patients with low/high FAM30A and TIMD4 expression.

Previously, we had constructed TMAs for 380 GC patients. FISH assays were conducted to detect the expression of FAM30A and TIMD4 in TMA (Fig. 8A, 8B). Using Image-Pro Plus 6.0 software, we obtained the AO value of each point in TMA. AO values represent FAM30A and TIMD4 expression. Our results showed that FAM30A or TIMD4 expression was upregulated in tumor tissues compared with paired corresponding normal tissues (P < 0.001, Fig. 8C, 8D). According to the level of FAM30A or TIMD4 expression and GC patient survival outcome, we used the ROC curve of SPSS software to calculate the cutoff value of FAM30A or TIMD4 (FAM30A cutoff = 0.0025, TIMD4 cutoff = 0.0112). As shown in Fig. 8E and 8F, the overall survival (OS) of the low-expression FAM30A or TIMD4 group was considerably more prolonged than that of the high-expression group by Kaplan-Meier survival curves.

## Discussion

The main cause of death in patients with GC is tumor progression and distant metastasis. The occurrence and development of GC is a pathologically complex process that involves many oncogenes and proto-oncogenes, such as mRNA, lncRNA, and miRNA 15,16. Therefore, there is an urgent need to discover new molecules related to GC metastasis to provide potential therapeutic targets for GC.

In our study, we used the WGCNA method to construct 18 coexpression modules to investigate metastasis-associated modules of GC. Our results showed that one module (the MEyellow module) was significantly associated with GC metastasis. Then, we selected the MEyellow module for further analysis. Through GO and KEGG analysis, DmRs and DlncRs in the MEyellow module were strongly enriched in such activities as opsonin binding, complement binding, integrin complex, protein complexes involved in cell adhesion, alpha-beta T cell activation and differentiation, cytokine-cytokine receptor interactions, and viral protein interactions with cytokines and cytokine receptors. In addition, these DmRs and DlncRs were also involved in immune function and cancer-related signaling pathways.

These DmRs and DlncRs in the MEyellow module were used to construct coexpression networks. Then, 7 DmRs (IL16, CD48, CCL19, SELL, BLK, LY9, and TIMD4) and 1 DlncR (FAM30A) that had higher connections with other nodes were considered hub nodes. These hub nodes might play important roles in the mechanism governing the metastasis of GC. Simultaneously, we conducted survival analysis using DmRs and DlncRs in the MEyellow module by the Kaplan-Meier method. The results illustrated that 5 DmRs (TIMD4, CETP, KRT27, PTGDS, and LRRC26) and 1 DlncR (FAM30A) were significantly associated with the OS of GC patients. Then, we used GC cell lines and tissues for validation. TIMD4, CETP, KRT27, PTGDS, and FAM30A were successfully validated, exhibiting higher expression levels in GC cell lines and tissues compared with GES-1 cells and normal tissues, respectively. However, LRRC26 exhibited the opposite pattern.

Based on previous bioinformatic analysis results, we found that FAM30A and TIMD4 were hub nodes and survival-associated biomarkers. To further investigate the clinical significance of FAM30A and TIMD4 in GC, we used FISH assay to detect their expression levels in TMA. Through these results, we concluded that FAM30A and TIMD4 were closely related to GC metastasis and could predict the prognosis of gastric cancer.

FAM30A is an lncRNA embedded in the immunoglobulin heavy locus on chromosome 14 and has antisense orientation to IgH gene fragments 17. FAM30A participates in various immune pathways related to the vaccine response and is related to the expression of immunoglobulin genes located in its genomic vicinity 17. FAM30A is highly expressed in B cells and participates in vaccine-induced responses. FAM30A participates in the immune response of inflammation, which is accompanied by B cell activation and immune-related gene changes 17. Although FAM30A is involved in the regulation of immune functions, FAM30A has not been studied in cancers. TIMD4 (also known as TIM4) is the main representative of T cell immunoglobulin and mucin domain (TIMD) family genes, which encode receptors for phosphatidylserine and participate in the phagocytosis of apoptotic cells by macrophages 18,19. TIMD4 recognizes phosphatidylserine, which is essential to effectively eliminate apoptotic cells and prevent autoimmunity 20. Previous studies reported that NSCLC patients with high TIMD4 expression had a poor prognosis 21. TIMD4 was overexpressed in renal cell lines, and the high expression of TIMD4 was closely related to a short progression-free survival time 22. TIMD4 promoted the growth of colorectal cancer by activating angiogenesis and recruiting tumor-associated macrophages through the PI3K/AKT/mTOR signaling pathway 23. In addition, TIMD4 could be detected in histiocyte sarcoma, histiocyte and dendritic cell tumors, and Langerhans cell sarcoma 24. However, the role played by TIMD4 in GC has not been reported to date.

In this study, we used bioinformatic methods to identify FAM30A and TIMD4 as possible biomarkers for predicting gastric cancer metastasis. Then, we verified the expression of these genes in gastric cancer cells and tissues *in vitro.* In addition, we used GC TMA to analyze the correlation between their expression and prognosis. Our data indicated that high expression of FAM30A or TIMD4 was worse than low expression regarding GC survival. These conclusions all suggested that FAM30A and TIMD4 could be effective markers for predicting the prognosis of GC and might also be potential therapeutic targets in GC.

## Figures and Tables

**Table 1:** The primers for qRT-PCR

Target		Sequence (5'-3')
FAM30A	F	TTGAATAGAGTAGTTCCTTGCGCTG
	R	GGCTACTTCACCCAGCTGTCTAG
TIMD4	F	TTGTCTGACTCCAACTGCCG
	R	TTGGCTGACTTCCTCGACAC
CETP	F	TCGTGTGCCGCATCACCAAG
	R	CCGTGATATCTGGGTAGCTG
LRRC26	F	CTGCTGCTGGACCACAACC
	R	AGAAGGCTCGCACATGCAC
PTGDS	F	CGGCTCCTACAGCTACCG
	R	CAGCGCGTACTGGTCGTA
KRT72	F	GTGGAGATTAACAGACGCACA
	R	GTTGTCCATTGACAGGACGAT
GAPDH	F	CTGACTTCAACAGCGACACC
	R	TGCTGTAGCCAAATTCGTTGT

**Table 2:** The number of DmRs and DlncRs in the 18 modules

Module color	All number	DmRs	DlncRs
black	1282	1212	70
brown	376	372	4
lightgreen	237	192	45
grey60	1571	1518	53
blue	469	457	12
turquoise	127	126	1
green	154	149	5
salmon	76	73	3
cyan	150	146	4
magenta	36	36	0
pink	32	28	4
midnightblue	55	51	4
lightcyan	59	55	4
greenyellow	42	41	1
purple	96	96	0
tan	112	111	1
red	71	71	0
yellow	44	42	2

**Table 3:** DmRs and DlncRs with more than 15 connections in the co-expression network in yellow module

Gene name	Degree	Biotype
IL16	20	mRNA
CD48	19	mRNA
CCL19	17	mRNA
SELL	17	mRNA
BLK	17	mRNA
LY9	16	mRNA
TIMD4	16	mRNA
FAM30A	16	lncRNA
